# Cold Atmospheric Plasma for Selectively Ablating Metastatic Breast Cancer Cells

**DOI:** 10.1371/journal.pone.0073741

**Published:** 2013-09-11

**Authors:** Mian Wang, Benjamin Holmes, Xiaoqian Cheng, Wei Zhu, Michael Keidar, Lijie Grace Zhang

**Affiliations:** 1 Department of Mechanical and Aerospace Engineering, The George Washington University, Washington, District of Columbia, United States of America; 2 Department of Medicine, The George Washington University, Washington, District of Columbia, United States of America; Faculté de médecine de Nantes, France

## Abstract

Traditional breast cancer treatments such as surgery and radiotherapy contain many inherent limitations with regards to incomplete and nonselective tumor ablation. Cold atomospheric plasma (CAP) is an ionized gas where the ion temperature is close to room temperature. It contains electrons, charged particles, radicals, various excited molecules, UV photons and transient electric fields. These various compositional elements have the potential to either enhance and promote cellular activity, or disrupt and destroy them. In particular, based on this unique composition, CAP could offer a minimally-invasive surgical approach allowing for specific cancer cell or tumor tissue removal without influencing healthy cells. Thus, the objective of this research is to investigate a novel CAP-based therapy for selectively bone metastatic breast cancer treatment. For this purpose, human metastatic breast cancer (BrCa) cells and bone marrow derived human mesenchymal stem cells (MSCs) were separately treated with CAP, and behavioral changes were evaluated after 1, 3, and 5 days of culture. With different treatment times, different BrCa and MSC cell responses were observed. Our results showed that BrCa cells were more sensitive to these CAP treatments than MSCs under plasma dose conditions tested. It demonstrated that CAP can selectively ablate metastatic BrCa cells *in vitro* without damaging healthy MSCs at the metastatic bone site. In addition, our study showed that CAP treatment can significantly inhibit the migration and invasion of BrCa cells. The results suggest the great potential of CAP for breast cancer therapy.

## Introduction

Breast cancer is the second leading cause of cancer deaths in women. It is estimated that 232,340 new cases of invasive breast cancer will be diagnosed in the United States and 39,620 women will die of the disease in 2013, according to the American Cancer Society. Breast cancer exhibits an affinity to metastasize to bone, resulting in debilitating skeletal complications associated with significant morbidity and poor prognosis. Roughly, 85% of individuals eventually develop bone metastases in advanced breast cancer [1]. The growth of disseminated tumor cells in the skeleton requires tumor cells to inhabit the bone marrow wherein metastatic breast cancer cells colonize the skeleton and interrupt normal bone remodeling processes. Current breast cancer treatment options such as surgery and radiotherapy contain severe limitations with regards to nonselective and incomplete tumor ablation. Thus, new treatments which can completely and selectively ablate solid tumors and transient breast cancer cells and tissues while keeping surrounding healthy cells and tissues intact is highly desirable.

Plasma is an ionized medium that contains numerous active components including electrons and ions, free radicals, reactive molecules, and photons [2]–[4]. It can be categorized as either thermal or non-thermal plasma [4]. Thermal plasma has been widely used to modify material surfaces, which is generally conducted in a vacuum [5], [6]. Cold atmospheric plasma (CAP), a non-thermal plasma, shows high electron temperatures but very low gas temperatures due to a weak ionization rate [5]. Because of the varied mass of CAP particles and constituents, thermodynamic equilibrium of electron self-collision occurs much faster than equilibrium between electrons and larger particles, such as ions [6], [7]. Thus, the overall plasma temperature is much lower than the electron temperature, which is close to room temperature. As an emerging technique for biomedical applications, CAP exposure has been shown to be highly effective in germicidal and sterilization, wound healing, blood coagulation, material surface modifications and crosslinking and treatment of various diseases, including cancer [8]–[14]. In particular, CAP can potentially offer a minimally-invasive surgical approach allowing for specific cancer cell or tumor tissue removal without influencing surrounding healthy cells and tissues, thus making it a promising technology for cancer therapy. Two possible underlying mechanisms for CAP's high selectivity towards cancer cells can be attributed to: (1) complex CAP composition and (2) the different nature of cancer cells and normal cells. Firstly, the variable cellular effects of CAP may be explained by the complex chemical composition of the CAP plume governed by the way the CAP is generated. Its presence can promote specific chemical reactions between charged particles and living cells triggering intracellular biochemical reactions that would elicit desired breast cancer therapeutic effects [15]. For example, reactive oxygen species (ROS, such as O, OH) are believed to be a major reason for cancer cell lysis [16] and bacterial inactivation [17], although the contribution of each type of the reactive species is still not well understood. In addition, the selective effects of CAP may be attributed to the significant difference of cancer cells and normal cells, thus rendering cancer cells more susceptible to CAP [18]. The objective of this research is to investigate a CAP-based therapy for the first time to selectively ablate metastatic breast cancer cell in metastatic bone site.

Traditionally, CAP has been developed to kill cells by utilizing deposition of different ROS active particles and excited radical, including UV photons [19]. Low dose of cold plasma treatment may have little to no effect on cancer cells, while high doses may ablate both cancer cells/tumor tissue and healthy cells/tissues. In this study, we will optimize CAP treatment parameters to selectively kill human metastatic breast cancer (BrCa) cells without minimally influencing healthy human bone marrow mesenchymal stem cells (MSCs).

For this purpose, three key CAP factors (i.e. treatment condition, exposure time, and treatment frequency) were tuned and evaluated. Firstly, we evaluated a series of CAP treatment conditions' (such as generator's output voltage and resistance) influence on MSC viability *in vitro*. After selecting the best treatment condition (i.e., minimum negative influence on MSCs), human metastatic MDA-MB-231 BrCa cells and MSCs with the same cell density were separately treated for 0, 30, 60, 90, and 120 s under this condition. Furthermore, BrCa cells and MSCs were cultured for 1, 3 and 5 days with daily CAP treatments (0, 30, 60 and 90 s). Cell growth and migration under designed CAP treatment were compared and evaluated.

## Materials and Methods

### BrCa cell and MSC culture

Human metastatic MDA-MB-231 BrCa cells (ATCC) were cultured in a complete media comprised of Dulbecco's Modified Eagle Medium (DMEM, Gibco) supplemented with 10% fetal bovine serum (Atlanta Biologicals) and 5% penicillin streptomycin solution (Invitrogen) and cultured under standard cell culture conditions (37°C, a humidified, 5% CO_2_/95% air environment).

Primary MSCs were derived from healthy consenting donor's iliac crest (Female; Age: 27). They were obtained at Tulane University under an IRB-approved protocol with informed consent, distributed from the Texas A&M Health Science Center, Institute for Regenerative Medicine and thoroughly characterized. We had the fully executed Material Transfer Agreement that was needed to obtain the cells. This provides verification that we in fact did not obtain the cells from the donors themselves. MSCs (passage #3–6) were cultured in a complete media comprised of Alpha Minimum Essential Medium (α-MEM, Gibco) supplemented with 16.5% fetal bovine serum, 1% (v/v) L-Glutamine (Invitrogen), and 1% penicillin streptomycin solution and cultured under standard cell culture conditions as described above.

### CAP characterization

**Figure 1:**
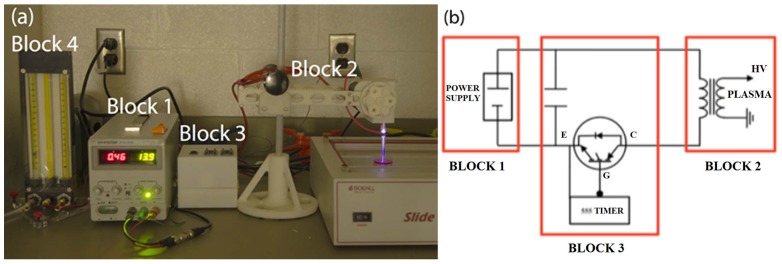
CAP set-up: (a) Configuration of CAP generation device. (b) electrical circuit diagram.

**Figure 2:**
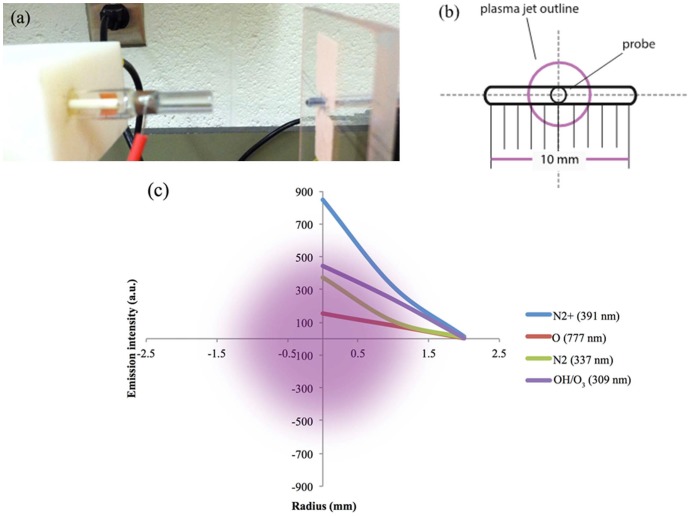
Radial measurement of helium plasma jet: (a) plasma tube aligned with optical probe (b) schematic diagram of holder and probe; (c) intensity of major species drop along radius.

The CAP device can produce diverse chemically active species. In our study, the CAP device consists of four blocks (Figure 1). Block 1 is a DC power supply 60 V/6A (SPS-606 Insteak). Block 2 is configured with a centrally powered electrode 1 mm in diameter and a ground outer electrode wrapped around a 4.5 mm diameter quartz tube which is the part of cold plasma production. Block 2 mainly controls the positional three degrees of freedom, allowing for rotational, vertical, and horizontal translation of the jet. Block 3 is made up of a capacitor, (VISHAY SPRAGUE-TVA1346-E3-100 μF, 100 V, axial) a transistor (STGW38IH130D, 33 A-1300 V-very fast IGBT), and a LM555 timer. LM555 timer contains two capacitors and two adjustable potentiometers R_A_ and R_B_. Block 4 is the helium gas supply. The helium flow rate was set to approximately 4.6 L/min. Electrical measurements were conducted with a Tektronix TDS3014C Digital Phosphor Oscilloscope. Optical measurements were made with a 100 µm probe 3 cm away from the front of the nozzle (Figure 2a). In order to measure the radius of the plasma jet, a holder with a horizontal trench (Figures 2a and 2b) was used to move the probe horizontally. Spectra of the jet were recorded at 0, 1, and 2 mm from the jet center. In addition, a radical experiment was conducted to measure the radius of the plasma jet.

### MSC and BrCa responses under different CAP treatment conditions and exposure time

MSC and BrCa cells were seeded at a density of 50,000 cells/well on two 48 well-plates, respectively. 300 µl of media was added into each well and all of the cells were cultured for 24 hours before CAP treatment. The distance between the CAP nozzle and plate bottom was 3 cm during all treatment conditions. All treatments were conducted at about 25°C (room temperature). After 30 s CAP treatment, media were replaced and cells were cultured for additional 24 hours. Firstly, MSCs were treated with different CAP conditions, including output voltage and resistance value as listed in Table 1. These parameters can alter the CAP discharge power and affect cell behaviors. The attached cell numbers after 24 hours of culture were compared to untreated controls.

**Table 1:** Different CAP parameter (output voltage and resistance value) effects for MSC growth *in vitro*.

Condition	Resistance A (Ω)	Resistance B (Ω)	Output Voltage (KV)	Effects
**1**	3000	3000	3.28	
**2**	3000	3700	3.28	−
**3**	3700	3000	3.28	−
**4**	3700	3700	3.28	+
**5**	3000	3000	3.68	+
**6**	3000	3700	3.68	−
**7**	3700	3000	3.68	−
**8**	3700	3700	3.68	−

Note: “+” means positive effects (i.e., non-inhibited or increased cell proliferation when compared to untreated group) for MSCs; “−” means negative inhibited effects (i.e., decreased cell proliferation when compared to untreated group) for MSC growth.

Two conditions (4 and 5 in Table 1) without negative inhibited influence to MSCs were prescreened, selected and applied to BrCa cells for 0, 30, 60, and 90 s. After the CAP treatments under the selected conditions, separately treated MSCs and BrCa cells were cultured for an additional 24 hours under standard cell culture conditions. After required culture time, the MTS assay reagent CellTiter96VR Aqueous One solution (Promega) was used to quantify attached cell number. In addition, a live/dead viability/cytotoxicity kit for mammalian cells (Molecular Probes) was used to evaluate cell viability after CAP treatment (condition 5 for 0, 30, 60, 90 and 120 s). Briefly, after 24 hours, each well was rinsed twice with phosphate buffer saline (PBS). A working solution of 4 µM ethidium homodimer (EthD-1) and 2 µM calcein AM was prepared as described previously [20]. After rinsing, 80 µL of working dye solution was added to each well and incubated at 37°C 30 min before imaging each well with a fluorescent microscope (Olympus BX 60). A mosaic image was collected in Image Pro^®^ to show and compare the distribution of live and dead BrCa cells and MSCs under 0, 30, 60, 90 and 120 s CAP treatments.

### BrCa and MSC proliferation under CAP treatments

BrCa and MSC were pre-seeded at 50,000 cells/well with 300 µL of media per well in a 48 well-plate and cultured for 24 hours. Similar to the previous CAP treatment section, CAP was used to treat both of the BrCa cells and MSCs. The optimal conditions for selectively ablating BrCa cells with minimally influence of MSCs were applied daily for 0, 30, 60 and 90 s. The cells were cultured in respective media for 1, 3, and 5 days.

At predetermined time points, MTS assay was used to quantify cell number. Briefly, cells were lift with Typsin EDTA, and added to a new 96 well plate with 100 µL of suspension per well. 20 µL of the MTS reagent was added to each well and incubated for 1 hour. Absorbance was measured at 490 nm using a UV-vis spectrophotometer.

### BrCa cell migration study after CAP treatment

In order to evaluate metastatic BrCa cell migration behavior after CAP treatment, the Matrigel Transwell Migration assay using a transwell chamber in conjunction with 8 mm membrane filter inserts was chosen. The pre-seeded BrCa cells were treated by CAP daily with different predetermined treatment times (0, 30, 60 and 90 s, condition 5). Then CAP treated cell were lifted and concentrated via centrifugation and further suspended in serum-free DMEM medium. 50,000 cells were added to the upper transwell chamber, whereas the lower chamber was filled with complete medium. After 24 hours of incubation in 5% CO_2_ at 37°C, BrCa cells which had migrated through the membrane to the lower surface were fixed and stained with a Differential Quik Stain Kit. The numbers of cells on the lower surface of the membrane were counted by a light microscope (10x).

### 2D wound healing assay of BrCa cells after CAP treatment

The wound healing assay was used to study BrCa cell migration velocity and cell interactions after 0, 30, 60 and 90 s CAP treatment. Treated BrCa cells (500,000 per dish) were seeded in a petri dish and allowed to adhere for 24 hours. Confluent monolayer cells were straightly scratched by a pipette tip, to simulate a wound environment, and then washed three times with PBS to remove cell debris and suspended cell. Fresh medium was added, and the cells were allowed to close the “wound” for 24 hours. Images of cells migrating into the wound space were captured at 0 to 24 hours via a confocal microscope. The rate of wound closure, BrCa cell migration velocity and migration distance of the first line of bottom of scratch in first 9 hours were calculated.

### Statistics

The cellular experiments were run in triplicate and repeated three times for each group. Data are presented as the mean value ± standard error mean (SEM) and were analyzed with student's t-test for pair-wise comparison. Statistical significance was considered at p<0.05.

## Results

### CAP characterization

**Figure 3:**
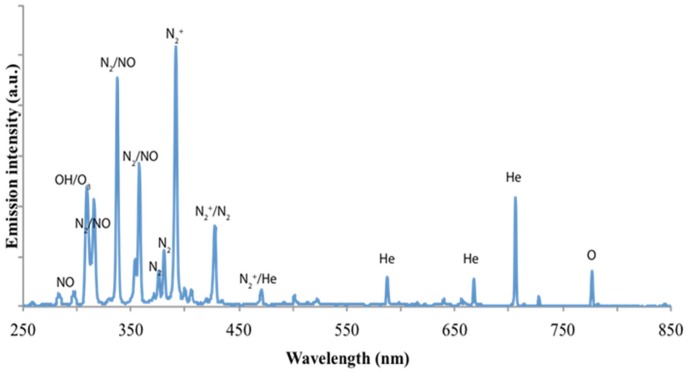
Emission spectrum of helium cold plasma at an output voltage of 3.68 kV.

Figure 3 shows a typical spectrum of helium CAP jet interacting with the ambient air. The identification of emission lines and bands were mainly according to Volotskova et al [21] and Georgescu et al [22]. In the significant range of 250–300 nm there are very weak emission lines, which are interpreted as NO [23]. In addition, the inactivation effect on bacteria by ultraviolet radiation is mostly related to biological damage caused by UV wavelengths of 200–280 nm. Thus it can be concluded that UV photons are not a major CAP species with our setup. In the wavelength range of 300–550 nm, their magnitudes are at most a few thousandths of the highest peak, N_2_^+^ (391 nm). The species lines between 300–500 nm are still undetermined [2], [4]. Other apparent CAP species were interpreted as He lines between 550–750 nm. Atomic oxygen (O) (emission lines at 777 nm) is believed to have significant biological effects, and was present in our generated CAP. The CAP jet is a highly complex environment which combines the comprehensive effects of different ions and neutrals. From the emission spectra data shown here, O (777 nm), OH/O_3_ (309 nm), N_2_^+^ (391 nm), N_2_ or NO lines (316, 337, 358, 427.5 nm) are the domain species of the spectra. Repeated measurements (not shown) were performed to confirm the initial results.

Radical measurement of the CAP jet was conducted with the holder shown in Figure 2. Helium flow rate was kept at 4.6 L/min. Input and output voltage was maintained at 11.8 V, and 3.76 kV, respectively. With a spectra of different positions recorded, the emission drops of major CAP species were plotted in Figure 2 (c). We can see that the radius of the jet is roughly 2 mm (i.e., ∼4 mm diameter).

In addition, it was observed that the plasma between the two electrodes formed by optical emission is much stronger than the plasma plume. Therefore, the spectra measured in our experiment may not be the entirely emission of the plasma jet. Furthermore, it should be pointed out that the probe placed in front of the jet may cause the perturbation of the helium plasma jet.

### Different BrCa cell and MSC responses under varied CAP conditions

Given its potential to interact with tissue or cells without a significant temperature increase, CAP holds great potential for minimally invasive cancer treatment. As mentioned previously, possible underlying mechanisms with regards to CAP's high selectivity towards cancer cells may be attributed to complex CAP compositions. In this study, we varied two CAP jet parameters (i.e., voltage and resistance) in order to achieve a desirable CAP composition and cellular responses.

**Figure 4:**
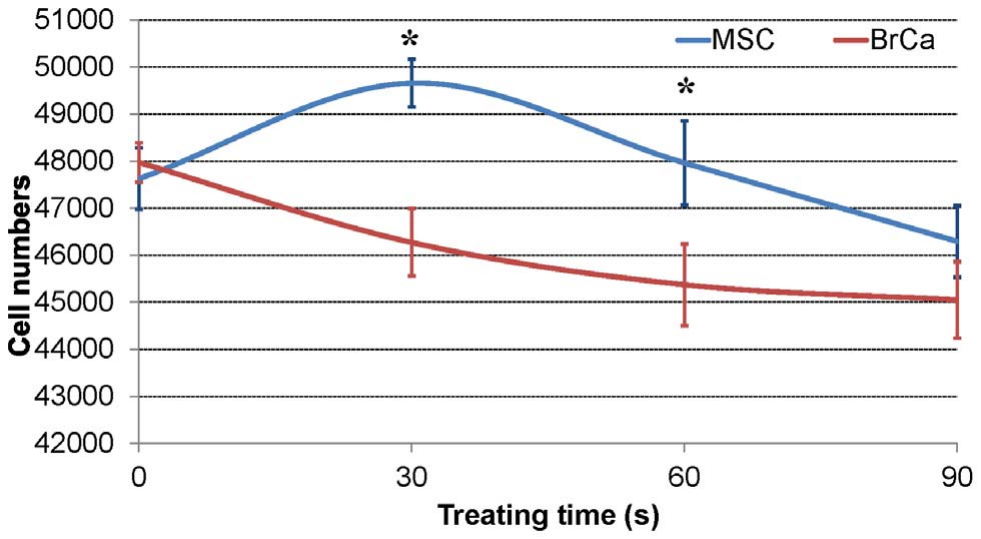
CAP effects on cell growth: MSC and BrCa cell growth under condition 5 (RA = 3000 Ω, RB = 3000 Ω, output voltage is 3.68 KV) in 0s, 30 s, 60 s, 90 s of CAP treatment. Data are mean ± SEM, n = 9; *p<0.05 when compared to BrCa cells under 30 and 60 s CAP treatments.

**Figure 5:**
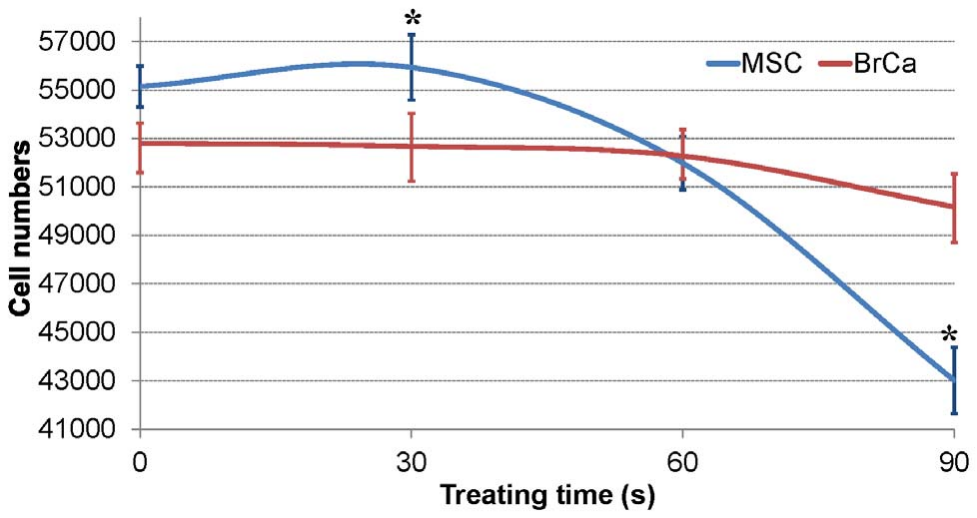
CAP effects on cell growth: MSC and BrCa cell growth under condition 4 (RA = 3700 Ω, RB = 3700 Ω, output voltage is 3.28 KV) in 0 s, 30 s, 60 s, 90 s of CAP treatment. Data are mean ± SEM, n = 9; *p<0.05 when compared to BrCa cells under 30 and 90 s CAP treatments.

Table 1 shows that different CAP voltage and resistance conditions lead to either a positive or negative effect on healthy MSCs. The 'positive effect' means non-inhibited or increased cell growth after CAP treatment. In other words, MSC can keep and improve their proliferation after the treatment. ‘Negative’ means a decreased cell growth number and changed morphology after CAP treatment. Among all conditions, conditions 4 and 5 reveal desirable positive effects on MSCs. Moreover, Figure 4 shows the selective effect of CAP treatment on MSC and BrCa cells under the condition 5 (RA = 3000 Ω, RB = 3000 Ω, and 3.68 KV output voltage). Within 30 s treating time, the MSC growth was greatly improved while BrCa cell growth was inhibited. In contrast, Figure 5 showed the effects of condition 4 on both MSCs and BrCa cells. Although MSC growth was greatly enhanced initially, there is not significant selectivity shown here. After 60 s treatment, both MSC and BrCa cell densities were decreased. Thus, the condition of RA = 3000 Ω, RB = 3000 Ω and 3.68 KV output voltage is the selected optimal treatment condition for the later experiments.

**Figure 6:**
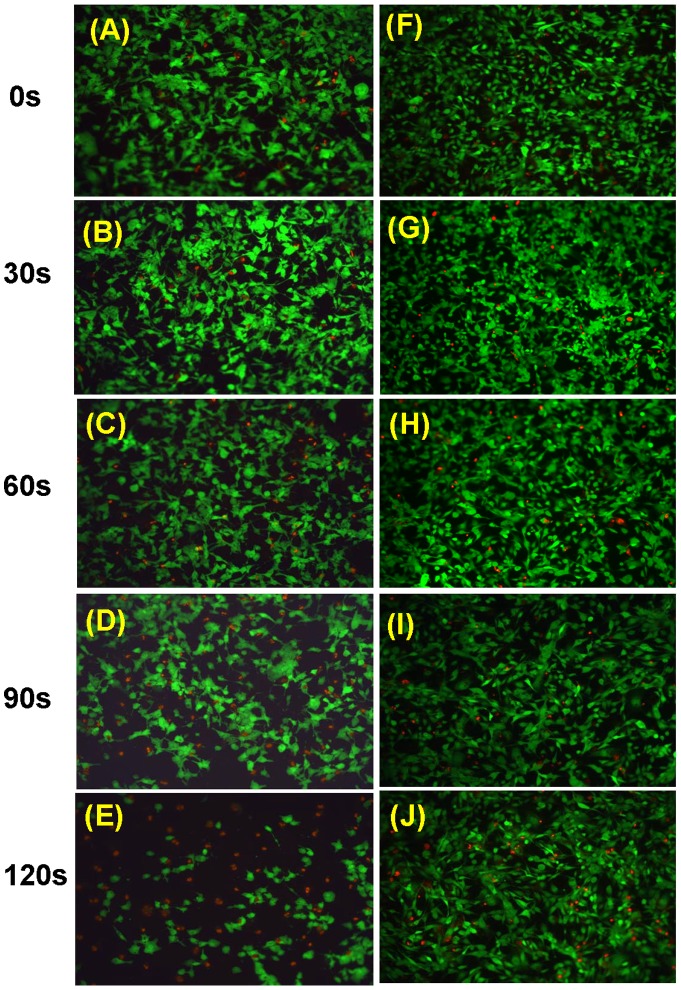
Fluorescence microscopy images. Live (green) and dead (red) BrCa cells (A–E) and MSCs (F–J) under 0, 30, 60, 90 and 120 s of CAP treatment.

Figure 6 illustrates the viability of metastatic BrCa and MSCs under 0, 30, 60, 90 and 120 s of selected CAP treatment condition 5. The images show typical and representative areas of the treated cells. Our results show that at short durations of CAP treatment (30 s), MSCs grew well when compared to both controls and the same treated BrCa cells. After 60 s of CAP treatment, significant differences between BrCa and MSC responses were observed. Greater amounts of metastatic BrCa cells were detached and lysed by CAP while MSCs kept growing normally with fewer dead cells (Figures 6C–E and 6H–J). Moreover, our results demonstrated that, for the first time, CAP treatment may selectively kill BrCa cells while leaving MSC growth unaffected. Although the underlying mechanism is not completely clear, we believe that a unique ROS (plasma killing species) and reactive nitrogen species (RNS, plasma healing species, such as NO, N_2_, N_2_^+^) environment created by CAP may contribute to the adverse and beneficial cell responses as shown in our study.

**Figure 7:**
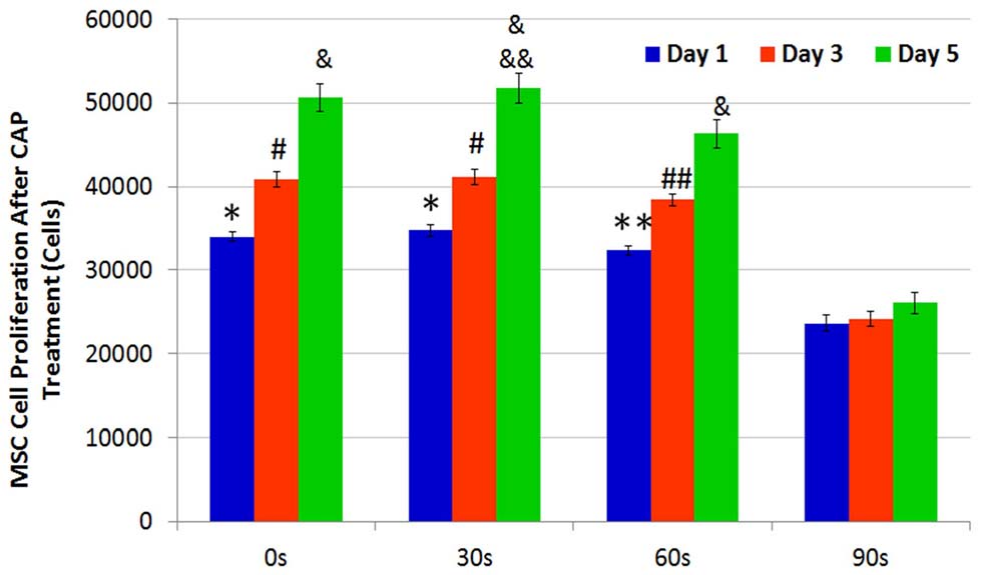
CAP treated MSC proliferation. MSC 1, 3 and 5 day proliferation under different daily CAP treatments. Data are mean ± SEM, n = 9; *p<0.05 and #p<0.01 when compared to 60 and 90 s daily treatment after day 1 and day 3; **p<0.05 and ##p<0.01 when compared to 90s treatment after day 1 and day 3; &p<0.01 when compared to 90 s daily treatment after day 5 and &&p<0.05 when compared to 60s daily treatment after day 5.

**Figure 8:**
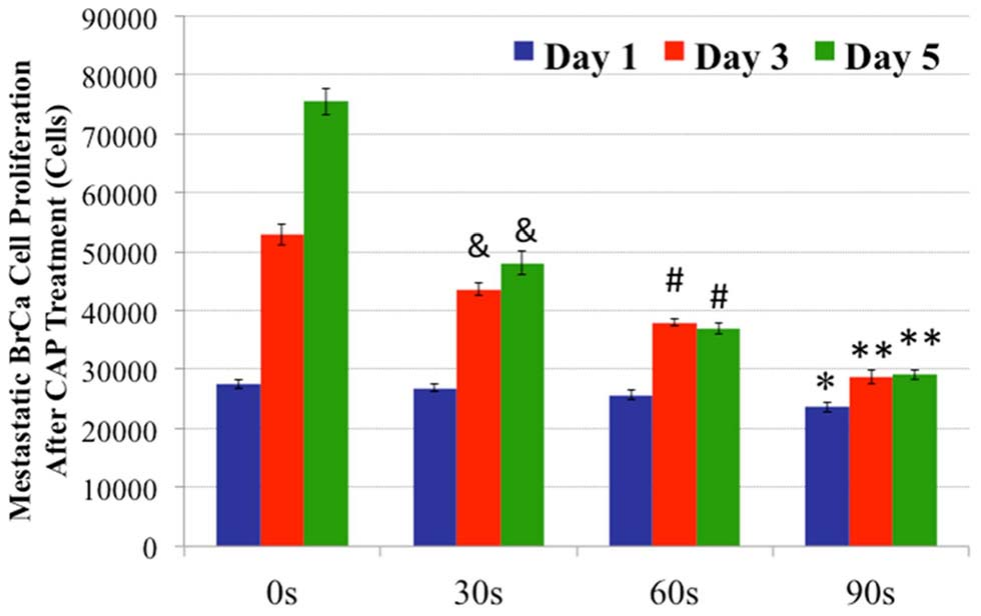
CAP treated BrCa cell proliferation. Significantly inhibited BrCa cell 1, 3 and 5 day proliferation under different daily CAP treatments. Data are mean ± SEM, n = 9; *p<0.01 when compared to 0 s and 30 s daily treatment after day 1; **p<0.01 when compared to all other samples after day 3 and day 5. #p<0.01 when compared to 0 s and 30 s daily treatment after day 3 and day 5; and &p<0.01 when compared to untreated samples (0 s) after day 3 and day 5.

Cell adhesion and viability data after different CAP treatments showed that the CAP device's voltage, resistance, and treatment duration played a significant role in modulating different cellular responses. Furthermore, in addition to single CAP treatments, MSCs and BrCa cells were periodically exposed to daily CAP treatment for up to 5 days. Both MSC and BrCa cell proliferation during the daily CAP treatments were evaluated and compared. The results of 1, 3, and 5 day MSC proliferation studies are shown in Figure 7. Under short (30 s and 60 s) daily CAP treatments, MSCs remained intact and proliferated well when compared to untreated controls (0s treatment). Under 90 s daily treatment, MSC cell density was lessened when compared to short term CAP treatment. In contrast, Figure 8 reveals a significantly different BrCa proliferation profile under the same daily CAP treatment regimen. Cancer cells were more sensitive to CAP when compared to untreated BrCa cells (0 s), all CAP-treated BrCa cells showed a significant inhibited proliferation rate after 3 and 5 days. Especially after 60 s and 90 s daily CAP treatment, cancer cells virtually stopped expanding. According to the results, selective ablation time for significantly lysing BrCa with the smallest possible effect on MSC is between 30–60 s.

### Inhibited BrCa cell migration after CAP treatment

**Figure 9:**
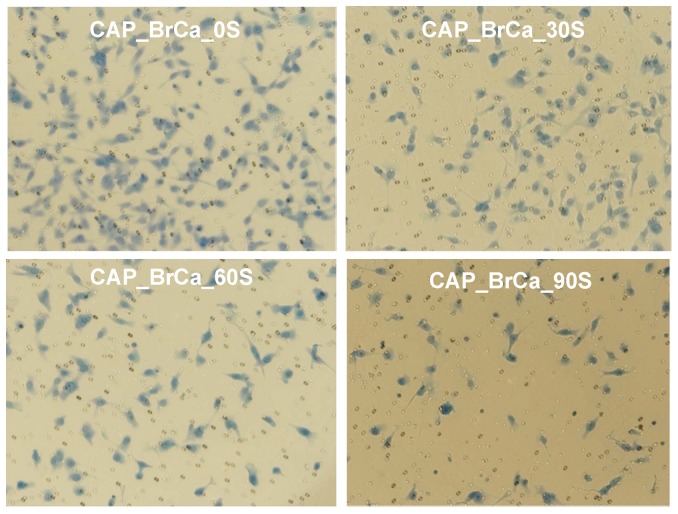
BrCa cell migration via Transwell Migration assay. CAP treatment decreased invasion of BrCa in matrigel transwell. Cells were treated with CAP in 0 s, 30 s, 60 s and 90 s. CAP treatment influenced invasion of BrCa cell in matrigel transwell.

**Figure 10:**
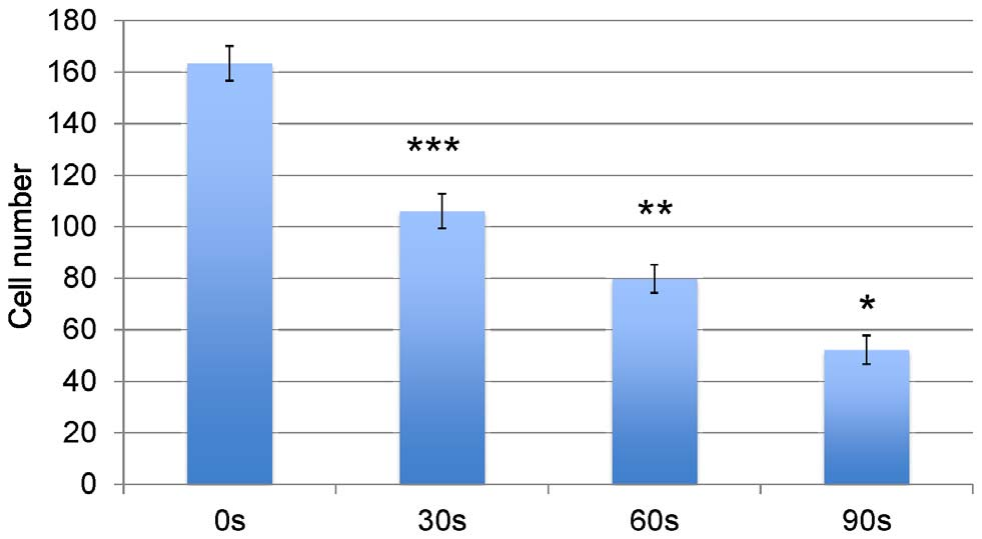
Quantified BrCa cell invasion in matrigel transwell. Data are mean ± SEM, n = 9; *p<0.001 when compared to all other treatments. **p<0.01 when compared to 0 s, and 30 s daily treatment. ***p<0.001 when compared to 0 s controls.

In addition, we investigated the CAP effects on the metastatic BrCa cell migration behavior via a transwell migration assay. Cell migration and invasion were significantly decreased in all three CAP treated BrCa cell groups (Figures 9 and 10). The number of migrated 90 s CAP treated cells decreased by nearly 3.5 folds when compared with control. The number of migrated 60 s CAP treated cells was reduced by 2 folds when compared to control. These results exhibited that CAP play an important role in mediating migration of metastatic BrCa cells.

**Figure 11:**
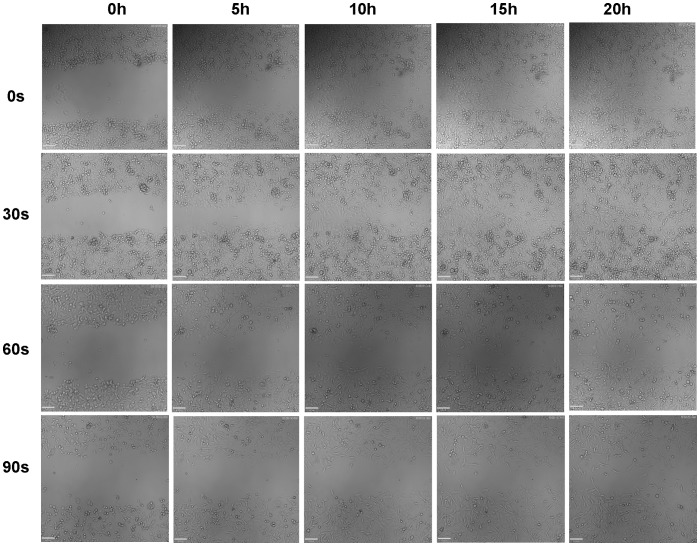
Wound healing assay: BrCa cells treated with different CAP time were captured every 5 hours.

**Figure 12:**
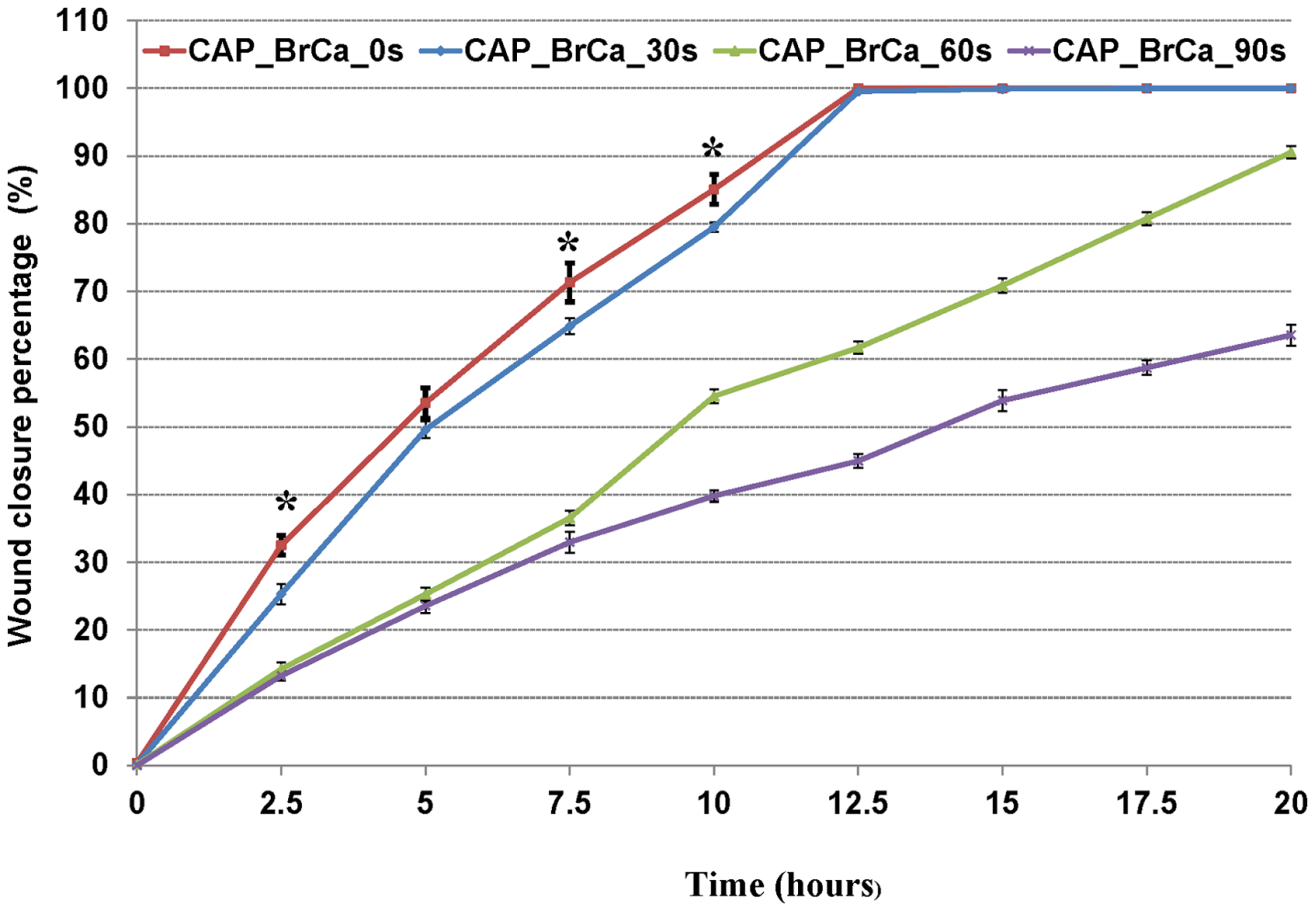
Representation of the rate of closure for each condition: untreated BrCa, CAP treated 30 s, 60 s, 90 s. Data are mean ± SEM, n = 9; *p<0.01 when comparing the untreated group with CAP treatment of 30 s, 60 s and 90 s.

**Figure 13:**
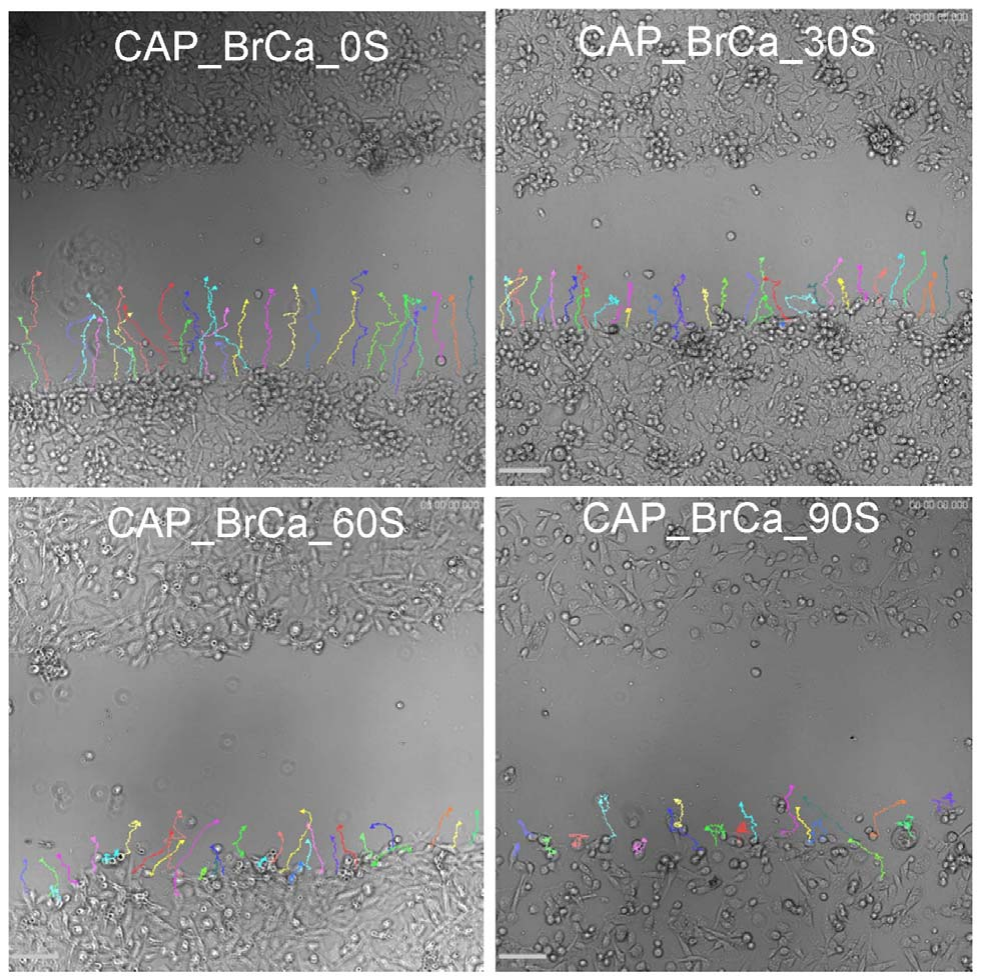
Representative microscopy images of BrCa migration pathway. Cell tracks of the first line of bottom of scratch for each condition in first 9 hours: untreated BrCa cells, CAP treated 30 s, 60 s, and 90 s.

**Figure 14:**
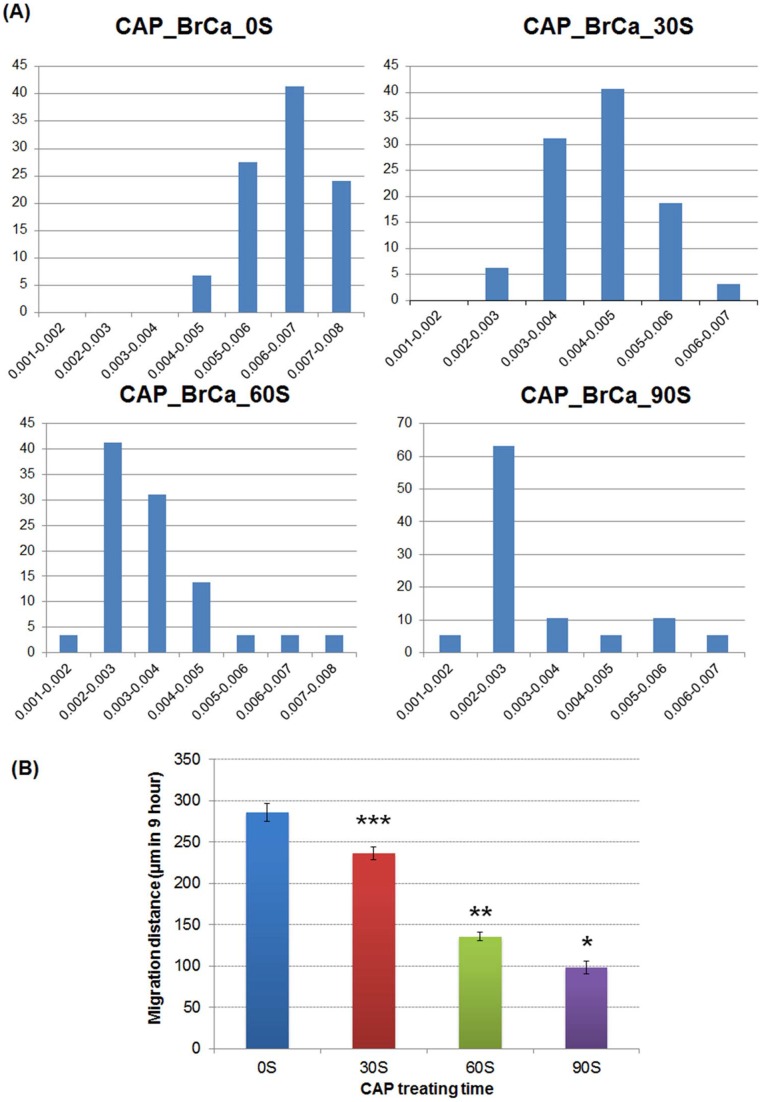
BrCa cell migration velocity after CAP treatment. (A) Cell velocity distribution of the first line of bottom of scratch for each condition in first 9 hours. X axis represents velocity distribution (µm/second) and Y axis represents cell percentage (%). (B) Quantification of average migration distance of BrCa cells under different CAP treatments in 9 hours. Data are mean ± SEM, n = 9; *p<0.001, **p<0.001 and ***p<0.001 when compared to all other treatments.

To further analyze cell migration and velocity after CAP treatment, a wound healing assay was performed to confirm the CAP inhibitory effects in BrCa cell migration. When started, the mean wound length was about 400 µm. After 20 hours incubation, the distance of untreated control was reduced to 0 (Figure 11). Consistent with the transwell migration results, CAP treated BrCa cells for 30, 60 and 90 s showed a significantly slow closure rate when compare to the control group. For 60s and 90s CAP treated BrCa cells, there was little to no wound closure after 20 h. 60 s CAP treated BrCa closed by 90.7%, whereas 90 s CAP treated BrCa only achieved 63.7% closure (Figure 12). Consistent with cell closure percentage data, Figure 13 shows the single cell migration tracks of the first line of the scratch showed the greatly slow migration of 30 s, 60 s and 90 s CAP treated BrCa cells after 9 hours migration. In addition, CAP treated cells presented a decrease in the number of high-velocity movements (Figure 14A), and exhibited a significant decrease in total migration distance (Figure 14B).

## Discussion

Tumor metastasis is the movement of tumor cells from a primary site to a secondary site (such as bone), which occurs in a series of steps including cancer cell migration, adhesion, proliferation, invasion and vessel formation, etc. All of those steps are regulated by complex mechanism including microenvironment interaction, gene expression and enzymes regulation. Our experiments demonstrated that through adjustment of resistance and output voltage of CAP jet, with the change of plasma emission intensity and power, a mild dose with suitable discharge power can be acquired to efficiently kill metastatic BrCa cells while keeping healthy MSC alive. Due to the observed significantly inhibited migration of BrCa cells under CAP treatment, one of the possible effects of moderate intensity and power plasma treatment may contribute to alter BrCa cell surface receptor functions [24] (such as integrin mediating cell adhesion and migration) [25]. Furthermore, the present research demonstrated that CAP treatment plays an important role in tumor therapy via immediate cell lyses [26]. Cell death in response to CAP treatment can be correlated to all aspects of the treatment condition, especially in the dose range. This is primarily a result of many likely factors including the presence of charged electrons, ROS, and RNS [27]. Furthermore, plasma-induced DNA damage may likely initiate cellular change. Although the possible underlying mechanisms of cell death induced by CAP remain unclear, previous research has elucidated several potential mechanisms for diverse cell responses under CAP [20], [28], [29]. For example, ROS in CAP is reported to play a large role in cancer cell and bacterial ablation [30]–[32]. CAP induced DNA-damage is purported to be attributed to ROS-induced DNA lesions caused by point mutations [33]. Recent reports also indicated that lipid peroxidation and oxidative DNA damage in E. coli was caused by intracellular ROS formation of CAP [34]. In our study, we observed that high doses of CAP treatment can significantly decrease cancer cell viability, which are believed to be mainly caused by ROS. In addition to DNA lesions caused by ROS, DNA precursor oxidation also has the potential to influence cell apoptosis [35], [36]. Also, it has been reported that CAP induced pH change of cell culture media may lead to cell apoptosis. However, our study showed that our medium pH change under the current CAP set-up was considered to be negligible. Furthermore, thermal effect on cells is virtually negligible in our study, which corresponds with our previous research [15]. Physically, CAP exposure can create temporal openings of the cell membrane, usually over a microsecond timescale, allowing for CAP specie transportation. This results in different cell responses [37]. In addition, low energetic UV photos may indirectly affect genes and cause cell death [37]. Moreover, since cancer cells proliferate much faster than normal cells, it has been reported that CAP can selectively target cancer cells by interfering with the mitotic cell cycle [18]. Thus, the observed selective effects of CAP in our study may be attributed to the significant difference in the distribution of BrCa cells and MSCs within the cell cycle, thus rendering cancer cells more sensitive to CAP treatment. Under fluorescence microscopy, specifically within the context of a live dead assay, we can clearly see that treated BrCa cells exhibited a significantly decreased viability with CAP treating.

Current results show that *in vitro* wound closure assay results were in line with the transmigration assay results. CAP treated BrCa cell wound areas close more slowly than untreated ones during a 20 h culture period. Interestingly, after 60 s and 90 s CAP treatment, when wounds reached close to full closure, BrCa cells in the first line of the bottom of the wound migrated in a horizontal direction instead of a vertical one. Consistent with the cell adhesion and proliferation studies, it is postulated that CAP exposure may also affect genes related to cell migration and metastasis, which play an important role in either horizontal (wound healing assay) or vertical (transmigration assay) directional migration.

Our current study focused on the initial interactions between CAP, MSCs and BrCa cells in order to optimize the parameters. The next step will be focused on devising an *in vitro* co-cultured model with a bone-tumor microenvironment and utilizing an *in vivo* model to further study CAP's effects. For future clinical applications, the CAP can be administered to any tumor locations in breast cancer patients via an endoscopic tube for endoscopic treatment. If the size of the solid tumor is large as is common in middle/late stage breast cancer patients, before conventional surgery, we expect that direct endoscopic exposure of CAP to the tumor can highly efficiently shrink the size of tumor; after surgery removal procedure, CAP can be further employed to kill existing/remaining cancer cells and prevent further metastases.

## Conclusions

In summary, this study investigated CAP generation conditions (voltage, resistances, treatment duration and frequency) for selectively killing BrCa cells. BrCa cells were found to be more sensitive to these CAP treatments than MSCs under plasma dose conditions tested. It has been demonstrated for the first time that CAP can selectively ablate metastatic BrCa cells *in vitro* without damaging healthy MSCs. The results suggest the great potential of CAP to be highly selective towards breast cancerous cells, resulting in tumor remediation with near complete ablation while maintaining healthy cells and tissue intact.
